# Association between Hypothyroidism and Hyponatremia: An Evidence-Based Literature Review

**DOI:** 10.7759/cureus.114273

**Published:** 2026-08-10

**Authors:** Syeda Fatima

**Affiliations:** 1 Medicine and Surgery, Rajiv Gandhi University of Health Sciences, Kalaburagi, IND

**Keywords:** antidiuretic hormone, electrolyte disorders, euvolemic hyponatremia, hyponatremia, hypothyroidism, myxedema, serum sodium, thyroid hormone withdrawal, water homeostasis

## Abstract

Hypothyroidism has traditionally been considered a cause of euvolemic hyponatremia. However, because patients with hypothyroidism often have coexisting conditions that may independently contribute to hyponatremia, it can be difficult to determine whether hypothyroidism is the true underlying cause. This narrative review evaluated 13 retrospective and prospective clinical studies identified through a PubMed search to examine the relationship between hypothyroidism and hyponatremia. Although several pathophysiological mechanisms have been proposed, most studies have failed to demonstrate that hypothyroidism directly causes hyponatremia. Studies reporting an association generally found hyponatremia to occur primarily in patients with severe hypothyroidism, particularly those with myxedema, whereas uncomplicated hypothyroidism was rarely associated with hyponatremia. Similarly, hypothyroidism accounted for only a small proportion of patients presenting with hyponatremia. As most of the available evidence is retrospective, these findings should be interpreted with appropriate caution. Overall, the available clinical evidence does not consistently support a causal relationship between hypothyroidism and hyponatremia in uncomplicated hypothyroidism. When evaluating patients with hyponatremia, hypothyroidism should not be presumed to be the underlying cause, and alternative etiologies should continue to be considered.

## Introduction and background

Hyponatremia is one of the most frequently encountered electrolyte abnormalities in clinical practice and is defined as a serum sodium concentration below 135 mEq/L [1]. Clinical manifestations vary from mild symptoms, such as nausea and malaise, to severe neurological complications such as coma and permanent neurological dysfunction [1]. Hyponatremia has a broad differential diagnosis, including the syndrome of inappropriate antidiuretic hormone (ADH) secretion, primary adrenal insufficiency, hypothyroidism, chronic heart failure, chronic kidney disease, liver cirrhosis, hypovolemia, and primary polydipsia [1].

Hypothyroidism has traditionally been considered a cause of euvolemic hyponatremia [2]. However, because the evaluation of hyponatremia relies on the assessment of volume status, distinguishing hypovolemia from euvolemia can be challenging in clinical practice [3]. Consequently, it is often difficult to determine whether hyponatremia is directly attributable to hypothyroidism or to coexisting conditions and other confounding factors [2].

This narrative review examines retrospective and prospective clinical studies evaluating the association between hypothyroidism and hyponatremia to determine whether the available evidence supports a causal relationship.

## Review

Materials and methods

This narrative review was undertaken to evaluate the association between hypothyroidism and hyponatremia. A literature search was performed in PubMed on May 31, 2026, using the search terms "hypothyroidism" AND "hyponatremia." The reference lists of relevant articles were also manually screened to identify additional eligible studies.

Retrospective and prospective clinical studies published in English evaluating the relationship between hypothyroidism and serum sodium concentration were included, whereas case reports, animal studies, conference abstracts, and studies without primary clinical data were excluded. Titles and abstracts were screened by the sole author, followed by full-text review of potentially eligible articles. Eligible studies were categorized into three groups: (1) hypothyroid populations, (2) acute hypothyroidism following thyroid hormone withdrawal, and (3) hyponatremic populations evaluated for hypothyroidism. A total of 13 clinical studies met the inclusion criteria and were included in the review.

As this was a narrative review, a formal PRISMA study selection process and meta-analysis were not performed.

Proposed pathophysiology

**Figure 1:**
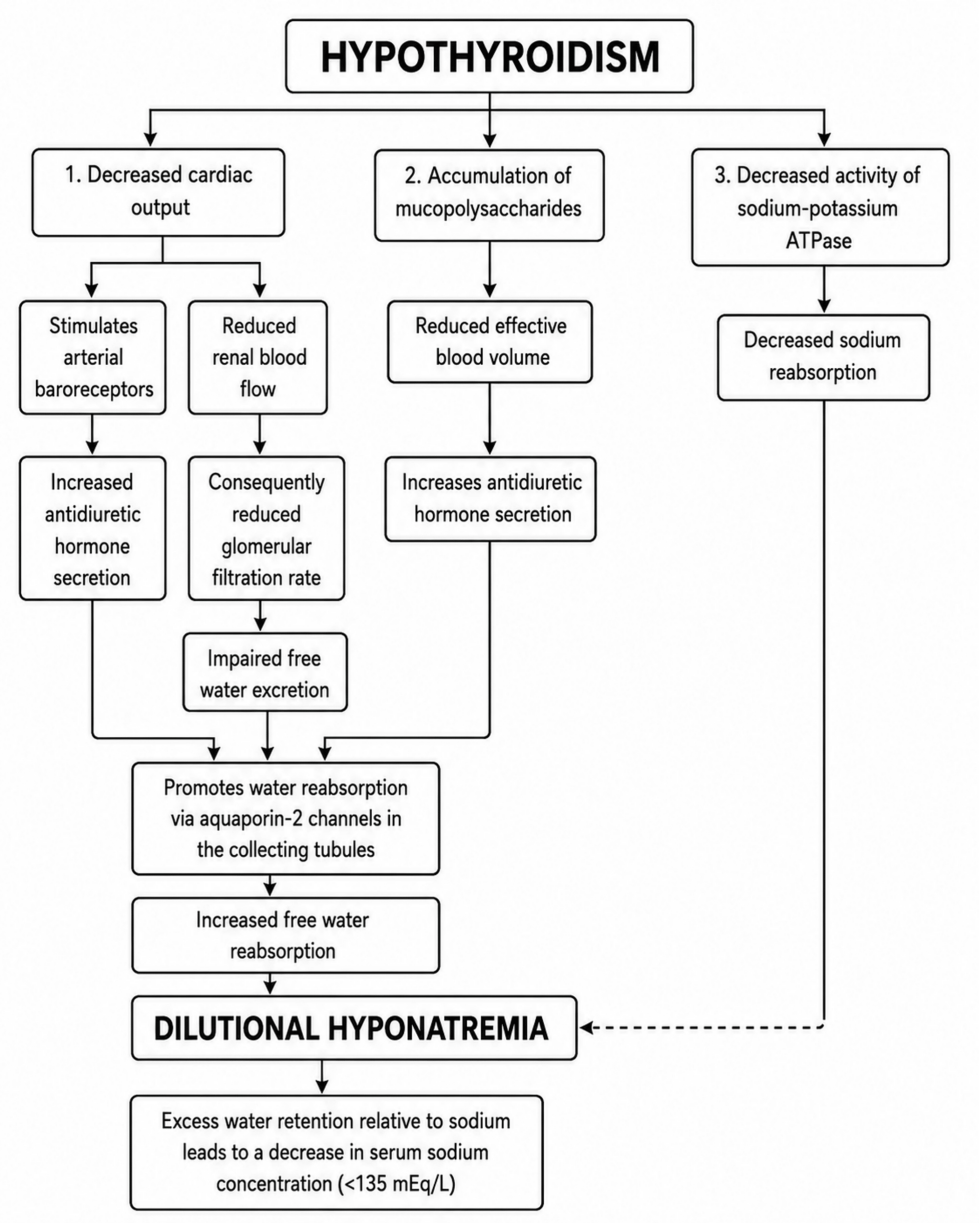
Proposed pathophysiological mechanisms linking hypothyroidism to hyponatremia Hypothyroidism decreases cardiac output and effective circulating volume, increasing antidiuretic hormone secretion, impairing free water excretion, and reducing sodium reabsorption, which may contribute to hyponatremia. ATPase, adenosine triphosphatase. Figure 1 was created using ChatGPT (OpenAI, San Francisco, CA, USA); the content and scientific accuracy of the figure were reviewed and verified by Syeda Fatima.

Several mechanisms have been proposed to explain the pathogenesis of hyponatremia in hypothyroidism [2]. Hypothyroidism is associated with decreased cardiac output and increased peripheral vascular resistance, which may stimulate arterial baroreceptors and result in increased ADH secretion [2,4]. In addition, interstitial accumulation of mucopolysaccharides within soft tissues may reduce the effective circulating blood volume, further promoting ADH release [2]. The resultant increase in ADH enhances water reabsorption through aquaporin-2 channels in the collecting ducts, potentially impairing free water excretion and contributing to dilutional hyponatremia, particularly when water intake exceeds physiological requirements [2]. Furthermore, the reduction in cardiac output may decrease renal blood flow and glomerular filtration rate, thereby impairing free water excretion [2-6]. Hypothyroidism has also been proposed to reduce Na⁺/K⁺-ATPase activity, potentially decreasing renal sodium reabsorption and further contributing to hyponatremia [2]. The proposed mechanisms are summarized in Figure 1.

Clinical studies

Various studies have investigated the relationship between hypothyroidism and hyponatremia; however, most have failed to establish a consistent causal association. The findings of the included studies are summarized in Table 1.

**Table 1:** Summary of clinical studies evaluating the association between hypothyroidism and hyponatremia. ED, emergency department; GFR, glomerular filtration rate; RAI, radioactive iodine; TSH, thyroid-stimulating hormone.

Study	Design	Population (n)	Key finding relevant to serum sodium-thyroid relationship
Serum sodium in hypothyroid populations			
Chu et al. [7]	Nationwide population-based retrospective	3,140 index patients with coexisting hypothyroidism and hyponatremia	Coexistence is uncommon.
Croal et al. [8]	Single-center retrospective cohort	33,912 (hypothyroid vs. euthyroid)	No difference in prevalence of hyponatremia between hypothyroid (12.8%) and euthyroid (11.4%) patients.
Sun et al. [9]	Single-center retrospective chart review	10 ambulatory patients, TSH >100 mIU/L	None had serum sodium below the reference range despite severe hypothyroidism.
Baajafer et al. [10]	Single-center retrospective cohort	188 patients with primary hypothyroidism	Mild hyponatremia in 3.9%; no correlation between serum Na⁺ and TSH.
Montenegro et al. [11]	Prospective, pre/post thyroid hormone replacement	41 patients with primary hypothyroidism	All had reduced GFR; 22 had elevated creatinine, of whom 45% were hyponatremic vs. 21% of those with normal creatinine - suggesting renal impairment as a modifier.
Warner et al. [12]	Retrospective, newly diagnosed ambulatory	999 hypothyroid patients	Every 10 mU/L rise in TSH associated with only 0.14 mmol/L decrease in Na⁺ - statistically significant but not clinically meaningful.
Schwarz et al. [13]	Retrospective analysis of ED records	9,012 emergency department encounters	Higher prevalence of hyponatremia among patients with elevated TSH, but Na⁺ did not correlate with TSH as a continuous variable.
Acute hypothyroidism after thyroid hormone withdrawal			
Gergics et al. [14]	Prospective observational, pre/post levothyroxine	39 patients with differentiated thyroid cancer	5% developed hyponatremia during acute hypothyroidism; negative correlation between Na⁺ and TSH; decreased apelin levels implicated.
Dayrit et al. [15]	Single-center retrospective, pre/post RAI	30 patients with differentiated thyroid cancer	No significant correlation between TSH and Na⁺ pre- or post-RAI.
Hammami et al. [16]	Prospective cohort undergoing RAI scanning	212 acutely hypothyroid patients	Moderate hyponatremia in 1.9%; no significant association between TSH and Na⁺.
Vannucci et al. [17]	Retrospective, pre-thyroidectomy vs. day of RAI	101 patients with differentiated thyroid cancer	Serum Na⁺ significantly lower on the day of RAI compared with pre-thyroidectomy values.
Hypothyroidism in hyponatremic populations			
Nagata et al. [18]	Multicenter retrospective cross-sectional	71,817 patients	Higher prevalence of overt hypothyroidism among patients with more severe hyponatremia.
Katoch et al. [19]	Prospective observational (altered sensorium)	100 patients with hyponatremia	Only 8% had hypothyroidism and 2% had adrenal insufficiency; 48% drug-induced (mainly thiazides), 42% idiopathic.

Serum Sodium in Hypothyroid Populations

A nationwide population-based retrospective study evaluating the epidemiology of coexisting hypothyroidism and hyponatremia identified only 3,140 patients with concurrent diagnoses [7]. Similarly, a large retrospective cohort study involving 33,912 patients found no significant difference in the prevalence of hyponatremia between hypothyroid and euthyroid individuals [8]. Consistent with these findings, a chart review of 10 patients with severe hypothyroidism (thyroid-stimulating hormone (TSH) >100 mIU/L) found that none were hyponatremic, suggesting that uncomplicated hypothyroidism rarely causes hyponatremia [9]. Likewise, a retrospective study of 188 patients reported mild hyponatremia in only 3.9% of cases, with no correlation between serum sodium concentration and the severity of hypothyroidism [10]. A prospective study of 41 patients with primary hypothyroidism before and after thyroid hormone replacement found that 22 patients had elevated serum creatinine levels, of whom 45% had documented hyponatremia [11].

Studies reporting a positive association have done so with important qualifications. A retrospective study of 999 newly diagnosed hypothyroid patients demonstrated that every 10 mU/L increase in TSH was associated with only a 0.14 mmol/L decrease in serum sodium; although this inverse relationship was statistically significant, the magnitude of change was clinically negligible [12]. Similarly, another retrospective study using 9,012 emergency department records reported a higher prevalence of hyponatremia among patients with elevated TSH levels [13]. These findings suggest that clinically significant hyponatremia, when present, is more likely to occur in patients with severe hypothyroidism or myxedema. However, severe hypothyroidism is frequently accompanied by organ dysfunction, which may have influenced these observations.

Acute Hypothyroidism: Thyroid Hormone Withdrawal Studies

To further evaluate this relationship, several studies have examined serum sodium concentrations in patients with differentiated thyroid cancer who develop acute hypothyroidism following thyroid hormone withdrawal before radioactive iodine (RAI) therapy.

A prospective observational study of 39 patients with differentiated thyroid cancer followed patients after thyroidectomy but before initiation of thyroxine therapy and found that only 5% developed hyponatremia, with a negative correlation between serum sodium concentration and TSH [14]. A retrospective study of 30 patients evaluated before and after RAI therapy likewise found no significant correlation between TSH and serum sodium levels [15]. Consistent with these findings, a prospective cohort study of 212 acutely hypothyroid patients undergoing RAI scanning also found no significant association between TSH and serum sodium levels [16]. In contrast, a retrospective study of 101 patients reported significantly lower serum sodium concentrations on the day of RAI therapy compared with pre-thyroidectomy values [17]. Overall, these studies suggest that acute hypothyroidism induced by thyroid hormone withdrawal is not consistently associated with hyponatremia.

Hypothyroidism in Hyponatremic Populations

Conversely, several studies have investigated the prevalence of hypothyroidism among patients presenting with hyponatremia. A large multicenter retrospective study involving 71,817 patients reported a significantly higher prevalence of hypothyroidism among patients with severe hyponatremia, even after adjustment for confounding factors [18]. However, this association may reflect organ dysfunction accompanying overt hypothyroidism rather than a direct causal relationship. Another observational study of 100 patients with hyponatremia found that only eight had hypothyroidism, whereas two had adrenal insufficiency [19]. Overall, these findings suggest that hypothyroidism accounts for only a small proportion of patients presenting with hyponatremia.

Limitations

This review has several limitations. Most of the available evidence is derived from retrospective studies, and many included patients had coexisting conditions that may independently contribute to hyponatremia, making it difficult to determine whether hypothyroidism was the true underlying cause. In addition, differences in study populations, the severity of hypothyroidism, and the definitions of hyponatremia across studies may have influenced the reported findings. Finally, as this is a narrative review, the conclusions are based on the available published evidence and should be interpreted in the context of the limitations of the included studies.

## Conclusions

The historical teaching that hypothyroidism causes euvolemic hyponatremia may overstate an association that is not consistently supported by the available clinical evidence. Although several pathophysiological mechanisms have been proposed to explain how hypothyroidism may lead to hyponatremia, the clinical data suggest otherwise. This may be because, in routine clinical settings, patients usually have multiple comorbidities that may independently contribute to hyponatremia. Most studies could not establish a causal relationship between hypothyroidism and hyponatremia, while those that did primarily associated it with severe hypothyroidism or myxedema. Even among studies conducted in patients presenting with hyponatremia, the prevalence of hypothyroidism was limited. This challenges the traditional association between hypothyroidism and hyponatremia.

During the evaluation of hyponatremia, hypothyroidism should not be presumed to be the underlying cause. Clinicians should continue to evaluate for alternative causes of hyponatremia, particularly in patients with uncomplicated hypothyroidism.
